# Application of objective physical activity measurement in an antenatal physical activity consultation intervention: a randomised controlled trial

**DOI:** 10.1186/s12889-015-2548-x

**Published:** 2015-12-18

**Authors:** Sinead Currie, Marlene Sinclair, Dianne S. Liddle, Alan Nevill, Marie H. Murphy

**Affiliations:** Psychology, School of Natural Sciences, University of Stirling, Stirling, Scotland; Institute of Nursing and Health Research, Ulster University, Newtownabbey, Northern Ireland; Faculty of Education, Health and Wellbeing, University of Wolverhampton, Wolverhampton, England; Centre for Physical Activity and Health Research, Ulster University, Newtownabbey, Northern Ireland

**Keywords:** Physical activity, Pregnancy, Decline, Patterns, RCT, Intervention

## Abstract

**Background:**

Physical Activity (PA) during pregnancy has many health benefits, however, inactivity in this population is common and PA often declines with increasing gestation. PA consultations have been useful in promoting PA in the general population, however their use for addressing PA in pregnancy is unknown. This study aimed to examine if a theory-based intervention using PA consultations would reduce the magnitude of decline in objectively measured PA between the first and third trimesters of pregnancy.

**Methods:**

A RCT was carried out in an urban maternity unit in Northern Ireland between September 2012 and June 2013. 109 low-risk, primigravida pregnant women were randomised to a control (*n* = 54) or intervention group (*n* = 55). Intervention participants received three face-to-face individual PA consultations. Daily PA was measured in each trimester using seven day accelerometry. The study was approved by a NHS trust (12/NI/0036). PA data in counts per minute (CPM) were categorised into intensity using Freedson cut points and mean minutes of PA were compared between groups using repeated measures ANOVA with a sub-analysis stratifying participants per PA level in trimester one.

**Results:**

Intention to treat analysis was performed on data from 97 participants. Time in moderate, vigorous and moderate-vigorous intensity PA (MVPA) significantly declined between trimesters one and three in both groups (*P* < 0.001). There were no statistically significant differences in PA between groups in any trimester. Women in the intervention group who were less active in trimester one did not demonstrate a significant decline in MVPA throughout pregnancy (in contrast with the decline identified in the more active participants).

**Conclusions:**

The findings indicate that PA consultations were not effective in reducing the decline of MVPA in throughout pregnancy, however, women who were less active in trimester one and received PA consultations had a lesser decrease in MVPA. It is possible that pregnant women, specifically those who are more active at the start of pregnancy, have differing needs for PA behaviour change and maintenance, requiring more intense interventions than less active women.

**Trial Registration:**

Current Controlled Trials Register ISRCTN61829137.

## Background

Women who are physically active throughout pregnancy experience a range of benefits including reduced pregnancy related discomfort [1], reduced risk of gestational diabetes mellitus [2], improved sleep quality [3], greater quality of life and feelings of happiness [4] and reduced risk of caesarean section [5]. Physically active pregnant women also reduce their risks of long-term weight retention, obesity and related chronic diseases [6]. Within the non-pregnant population, it is recognised that a physical activity (PA) dose–response exists, where any increase in PA leads to additional health benefits [7]. This notion that ‘some is good, more is better’ is likely to hold true for the pregnant population but there are still considerable gaps in the evidence. Within the UK, healthy pregnant women without medical or obstetric complications are encouraged to engage in a minimum of 30 mins of moderate intensity PA at least five times a week [8].

In most countries only a small proportion of non-pregnant women meet the recommended levels of PA [9]. In pregnant women this proportion decreases further. For example, in Canada, Gaston and Vamos [10] reported only 23 % of women who classified themselves as pregnant met PA guidelines, a proportion which is echoed in the USA, Ireland, Spain, and Portugal [11–14]. Furthermore, PA declines over the course of pregnancy further reducing the proportion of women exposed to the concomitant health benefits [11, 15–17]. This evidence underlines the importance of developing interventions that will help to reduce such a decline. Sudden uptake of new exercise and fitness regimes during pregnancy are not recommended [8], instead; guidelines suggest a gradual increase in the amount of PA undertaken. Maintaining or gradually increasing PA levels during the course of pregnancy can be difficult for pregnant women, therefore it is important that women set achievable goals in order to prevent the usual decline and facilitate the health benefits derived from regular PA. A fundamental question therefore is how can women be encouraged and supported to engage in, or maintain existing PA levels during pregnancy?

A range of interventions have been designed and tested to influence antenatal PA. A systematic review [18] concluded that eight of the ten included studies demonstrated that women who received an intervention incorporating behaviour change techniques (BCT’s) were more active at follow-up than those randomised to control groups. The most commonly applied BCT’s in successful studies included goals and planning, shaping knowledge and repetition and substitution. However, these interventions relied upon self-report measurement which has been shown to have a weak correlation with objective PA measurement. One such objective measurmenet of PA, accelerometry, is regarded as more robust than self-report [19, 20] and its use to measure PA has become increasingly popular. In the pregnant population, these monitors can be slightly inaccurate for step count although they are still recognised as a valid and reliable measure of PA [21–23]. Use of objective PA measurement during pregnancy is still in its infancy, and some considerations need to be made to current protocols and recommendations to ensure accurate measurement of behaviour in order to adequately assess efficacy of any behaviour change intervention. Considerations including adjusted wear time criteria and potentially revised cut-points should be made when using accelerometry to measure PA in pregnant women.

One intervention which has been successful in changing PA behaviours in the non-pregnant population is short one-on-one PA consultations [24]. Such consultations incorporate BCT’s suited to an individual’s stage of change [25] and have been used in a range of sedentary and inactive populations, with notable success in diabetic patients. A review by Kirk et al. [25] found that physical activity significantly increased in PA consultation groups compared with controls. Furthermore, a large study by Fitzsimmons et al. [26, 27] reported success using PA consultations with healthy, low active adults. Consultations are relatively low cost, in comparison to structured exercise programmes, and are suitable for a wide range of individuals, regardless of existing fitness levels [25]. However, their applicability to the pregnant population has not been investigated. For this reason, and the limited contact time required (approximately one hour per consultation), this intervention could easily be tested and delivered in a pregnant population with minimal additional expense. Therefore, theory based PA consultations offer a potential intervention for addressing PA behaviour change in pregnant women.

The Medical Research Council (MRC) [28] as well as other researchers in the area of physical activity during pregnancy [29] recognise the importance of incorporating theory when designing complex interventions. A stage model, specifically relevant to pregnant women which can be incorporated into PA consultations is the Health Action Process Approach (HAPA) [30]. This model recognises and categorised individuals into one of three stages of change (pre-intenders, intenders and actors) as well as providing the theoretical constructs and behaviour change techniques (BCT’s) relevant to individuals in each stage. The HAPA specifically relates to PA during pregnancy as it recognises risk perceptions, outcome expectancies and self-efficacy as contributors to intention, all of which are barriers to antenatal PA cited by women in previous literature [31, 32]. Furthermore, the constructs related to those classed as ‘intenders’ include goal setting and planning, which were seen as successful behaviour change techniques in previous antenatal PA research.

Taking the above evidence into consideration, the aim of this study was to objectively measure the PA levels of a group of pregnant women who received standard antenatal care plus PA consultations, based on the HAPA, and to compare these to a control group receiving standard antenatal care alone to establish whether the addition of PA consultations reduced the magnitude of decline in PA between the first and third trimesters of pregnancy. It was hypothesised that women randomly allocated into the intervention group and receiving the PA consultations would reduce their PA levels to a lesser extent than the control group from trimesters one to three.

## Methods

### Study design

A pragmatic randomised controlled trial, known as the Active Pregnancy Profile (APP) Trial, was conducted at the Ulster Hospital, Maternity Unit, Dundonald, Northern Ireland. The study received institutional ethical approval from the university and the NHS trust ORECNI (12/NI/0036). Recruitment took place between September 2012 and June 2013.

### Participants

One hundred and nine (109) primigravida, healthy pregnant women were recruited between 8 and 15 weeks’ gestation (mean gestation at recruitment was 10.5 +/− 1.3 weeks) at routine antenatal booking appointments. Women with medical or obstetric complications or risks were excluded from participation (in line with American College of Obstetricians and Gynecologists (ACOG) guidelines [33]), i.e., contraindications to being physically active; multiple gestation; under 18 years old, BMI under 19.

### Recruitment and randomisation

Recruitment took place over a 9 month period between September 2012 and June 2013. At the end of each standard antenatal booking appointment, if a midwife deemed a woman eligible to participate in the APP trial according to the inclusion criteria, the researcher was invited into the appointment room to explain what the research entailed. Following this brief explanation, potential participants were invited to attend an information session where details of the trial were explained and written informed consent was provided by participants. Participants were randomised to the intervention or control groups using sealed opaque envelopes containing group allocation, generated by a statistician using a computerised random number generation programme. Envelopes were prepared by an independent researcher. The researcher in charge of recruitment was not blinded to group allocation however, all data provided by participants was anonymised, with an individualised participant number appearing on all documentation instead of participant name or identifiable information. All completed documentation, for example questionnaires and diaries, were placed in a brown envelope (provided in each pack) and handed to the maternity hospital receptionist, to ensure the researcher would remain blinded to the identity of the participants.

**Fig. 1:**
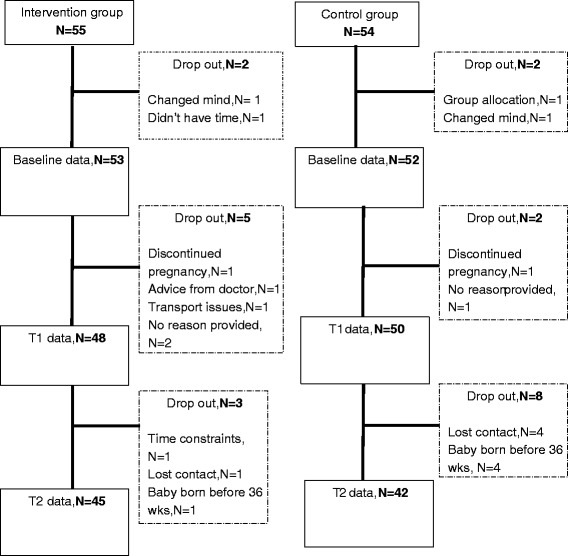
Flowchart of participant progress and drop out through the APP Trial

A total of 418 eligible women were invited to participate in this study, from which, 109 were randomised to the intervention or control group (for further information on recruitment see Currie et al. [34]). Of the 109 participants, four dropped out before providing baseline/trimester one information, seven dropped out before providing trimester two data and 11 dropped out before providing trimester three data, an overall drop out rate of 20 %. A flow chart of participant progression is shown in Fig. 1. Forty-seven women (87 %) in the intervention group attended at least two of the three consultations. Only participants who provided sufficient baseline/trimester one PA data (eight hours wear time on at least four days), and at least trimester two or three PA data were included in analyses, resulting in data from 97 participants (47 intervention and 50 control).

### Control group

Women randomised to the control group received standard antenatal care, as provided by the health trust. This included three hospital appointments and an additional eight community midwife/GP appointments. These appointments were scheduled to occur between eight and 14, 16 to 18, 18 to 20, 25, 28 to 30, 31 to 32, 34, 36, 38, 40 and 41 weeks’ gestation. In line with National Institute for Health and Care Excellence (NICE) guidelines [35], exercise was discussed with women at the first antenatal appointment (booking appointment). A description of these guidelines can be found in Table 1. There was no further mention of exercise at routine antenatal appointments during the course of pregnancy, according to NICE guidelines. Control group participants were asked to complete the same outcome measures as the intervention group, and at the same three time points; 12 to 15 weeks’, 20 to 22 weeks’ and 35 to 37 weeks’ gestation.

**Table 1:** Exercise advice provided to pregnant women as part of usual care, according to NICE guidelines [33]

At booking appointment (ideally by 10 weeks’ gestation), women should be provided with information regarding exercise, including pelvic floor exercises specifically:
• Pregnant women should be informed that beginning or continuing a moderate course of exercise during pregnancy is not associated with adverse outcomes • Pregnant women should be informed of the potential dangers of certain activities during pregnancy, for example, contact sports, high-impact sports and vigorous racquet sports that may involve the risk of abdominal trauma, falls or excessive joint stress, and scuba diving, which may result in fetal birth defects and fetal decompression disease.

### PA intervention

In addition to the standard antenatal care described above, women randomised into the intervention group received three individually tailored PA consultations (one per trimester) based on their individual stage of change according to the HAPA. The content of these consultations are described below and can be found in Table 2. These consultations occurred between 12 to 15 weeks’, 20 to 22 weeks’ and 32 to 34 weeks’ gestation and were delivered by one researcher fully trained in delivering PA consultations. The consultations were face-to-face lasting between 30 to 60 min and encouraged women to achieve 30 min of moderate intensity PA at least five times per week [33]. In accordance with the Royal College of Obstetricians and Gynaecologists (RCOG) [8], women who were previously engaging in none or very little moderate intensity PA were advised to begin with 15 min of moderate intensity PA three times per week, gradually increasing to 30 min four times per week. If they felt uncomfortable increasing this amount, they were encouraged to maintain at least 15 min of moderate intensity PA three times per week. Maintenance was encouraged for women who were already meeting this guideline. The Borg scale of perceived rate of exertion [36] was used to explain appropriate intensity (moderate equating to a scoring between 12 and 13 on a six to 20 scale). All four domains of PA (leisure time, occupational, household, commuting [37]) were encouraged as long as they were performed at moderate intensity. Activities that the participant enjoyed and had easy access to were encouraged e.g., walking, swimming and housework. In accordance with the HAPA [30], each consultation involved a review of the participant’s PA during the previous trimester and an assessment of the stage of change they were currently in using a stage of change question adapted from PA consultations by Loughlan and Mutrie [24]. This information then prompted a discussion about the frequency, intensity, time and type of PA the participant typically engaged in on a weekly basis. Depending upon PA engagement and stage of change category, the HAPA theoretical constructs relevant to that stage and PA consultation principles were delivered via BCT’s (Table 2 provides examples) [38]. For example, someone classed as an ‘intender’ received all BCT’s relevant to this stage; goal setting (behaviour), action planning, problem solving and unspecified social support.

**Table 2:** Theoretical components, behaviour change techniques applied and examples from PA consultations

HAPA stage	HAPA components	Behaviour change technique	Example
Pre-intender	Risk perceptions, Outcome expectancies, Task self-efficacy	Information from a credible source	Information from trained researcher
		information about health and emotional consequences	Information regarding benefits of PA during pregnancy
		pros and cons	Discussion of pros and cons of engaging in PA during pregnancy
		focus on past success	Discussion of activities engaged in previously which were enjoyed (pre-pregnancy or last trimester)
Intenders	Action planning, Coping planning, Coping self-efficacy	goal setting: behaviour	Set a personalised goal for the engagement in PA per week (SMART goal)
		action planning	Write a detailed plan of how, when and where the goal related PA will occur
		problem solving	Look ahead for events which may hinder goal success and think of ways to reduce this being a problem
		unspecified social support	Think about friends and family who can help with engagement in PA and achievement of goal
Actors	Recovery self-efficacy, Maintenance	review of behavioural goals	Discuss how well the participant met the previously set goal. Modify or continue with same goal.
		Action planning	Write a detailed plan of how, when and where the modified or continued goal related PA will occur
		goal setting: behaviour	Set a personalised goal for the engagement in PA per week (SMART goal)
		focus on past success	Discussion of activities engaged in previously which were enjoyed and engaged in (pre-pregnancy or last trimester)

### Outcomes

PA was objectively assessed at 12 to 15 weeks’ (baseline, classed as trimester one), 20 to 22 weeks’ (trimester two, following PA consultation two for the intervention group) and 35 to 37 weeks’ (trimester three, following PA consultation three for the intervention group) gestation, using an accelerometer (Actigraph model GT3X, Actilife, Pensacola, Florida, USA). Accelerometers were worn around the waist on an expandable elastic belt with the device placed over the right hip for all waking hours on seven consecutive days. The device was worn below the bump to avoid any effect of tilt. The hip was chosen as placement on the trunk of the body is classed as the most accurate in non-pregnant populations [39] and this is the most common placement in studies measuring PA in pregnant women, thus facilitating direct comparison. All participants were provided with the monitor by the researcher following the recruitment session, a PA consultation or a routine hospital appointment. Data were collected using a sampling interval of five second epochs, in order to gain as much information as possible. Raw data were analysed using mean counts per minute (CPM) and Freedson [40] cut points were applied to determine exercise intensity. CPM was calculated by the device accumulating the acceleration data into a certain number of activity CPM. Typically, the more CPM equates to a higher PA intensity. The published intensity cut-points by Freedson [40] categorised these CPM into time spent in specific PA intensities. Freedson [40] defined light intensity PA between 100–1952 CPM, moderate intensity PA as 1952–5724 CPM and vigorous intensity PA as over 5724 CPM. Criteria regarding wear time were defined as per previous research using accelerometers with pregnant women who indicate that wear time is significantly lower in this group [14, 22, 41, 42]. Non-wear time was defined as 60 min or more of consecutive zeros. Valid wear time was defined as at least eight hours a day, four days a week including at least one weekend day, which is lower than PA research with non-pregnant groups but reflects previous research with pregnant women. Accelerometer wear time with less than the above criteria were classed as missing.

Demographic data were collected at baseline including age, BMI, marital status, education employment and smoking status.

### Statistical analysis

The demographic characteristics of each group were analysed as means and standard deviations (SD) for continuous variables (age and BMI) and as a percentage for categorical variables (marital status, education, employment, smoking status). Groups were compared using independent t-tests for continuous variables and Chi-square or Fishers exact test for categorical variables. Analysis was performed using intention to treat (ITT), which required a full data set. Any missing PA data was dealt with using the Estimation Maximisation (EM) procedure as implemented in SPSS (version 21). The residuals of all PA intensities and CPM variables were checked for normality. The majority of residuals were classed as normal with a non-significant Kolmogorov-Smirnov result, with skew and kurtosis between one and minus one.

Repeated measures ANOVA were used to investigate any change over time between groups. This analysis assumed compound symmetry, if this assumption was violated, the significance value was based on the Huynh-Feldt epsilon adjustments. To further investigate whether the intervention effects differed between participants who were more active or less active, a sub-analysis was performed. A median split was applied to the trimester one MVPA in both groups (37.17 min), with participants categorised as less active (below median) or more active (above median). MVPA was log transformed in order to ensure normality. A repeated measures ANOVA was then performed with the trimester one split included as a between subjects factor. All analyses were performed using IBM SPSS statistics 21 with a level of significance set to less than 0.05.

## Results

Baseline (trimester one) characteristics of women in the control and intervention groups are shown in Table 3. All participants were non-smokers. At baseline (trimester one) there were no differences in demographics or PA between the intervention and control groups (Table 4).

**Table 3:** Trimester 1 (baseline) demographic in the intervention and control group

Characteristic	Intervention group	Control group	*p*-value
	*Mean (SD)*	*Mean (SD)*	
	*N = 47*	*N = 50*	
Age (years)	31.7 (4.1)	30.55 (4.9)	0.254
Weight (kg) at recruitment	69.7 (11.1)	71.0 (12.5)	0.593
Body Mass Index at recruitment	25.5 (3.3)	26.1 (4.9)	0.502
Gestation at recruitment (weeks)	10.56 (1.3)	10.41 (1.35)	0.569
	*N (%)*	*N (%)*	
Martial Status	*46*	*48*	0.569
Single	3 (7)	4 (8)	
Living with partner	15 (33)	11 (23)	
Married	28 (61)	33 (69)	
Highest educational qualification	*46*	*48*	0.473
Below secondary	0 (0)	2 (4)	
Secondary	9 (20)	13 (27)	
Tertiary	37 (80)	33 (69)	
Employment status	*45*	*48*	0.618
Employed	44 (98)	45 (94)	
Unemployed	1 (2)	3 (6)

**Table 4:** Physical activity data for intervention and control group in each trimester

	Intervention	Control
Trimester 1		
Accelerometer weartime (hrs per day)	13.4 (+/− 1.0)	13.3 (+/−1.0)
Accelerometer weartime (valid days)	6.7 (+/−0.9)	6.5 (+/−1.0)
Light intensity (mins per week)	112.0 (+/− 29.0)	110.0 (+/−35.8)
Moderate intensity (mins per week)	38.5 (+/−17.0)	37.0 (+/−14.2)
Vigorous intensity (mins per week)	2.6 (+/−4.6)	3.1 (+/−4.0)
MVPA (mins per week)	41.0 (+/−19.8)	40.0 (+/−15.6)
MVPA (mins per week) less active participants	26.1 (+/− 7.6)	28.2 (+/− 6.4)
MVPA (mins per week) more active participants	56.5 (+/− 16.2)	51.8 (+/− 12.8)
CPM	274.4 (+/−114.7)	274.3 (+/−96.1)
Trimester 2		
Accelerometer weartime (hrs per day)	13.1 (+/−1.1)	13.3 (+/− 1.0)
Accelerometer weartime (valid days)	6.5 (+/−1.0)	6.8 (+/−0.9)
Light intensity (mins per week)	112.3 (+/−24.8)	120.8 (+/− 35.6)
Moderate intensity (mins per week)	39.2 (+/− 17.4)	37.5 (+/−15.7)
Vigorous intensity (mins per week)	1.9 (+/−3.3)	1.7 (+/−2.6)
MVPA (mins per week)	41.1 (+/−19.0)	39.2 (+/− 16.4)
MVPA (mins per week) less active participants	30.96 (+/− 13.42)	29.4 (+/− 10.7)
MVPA (mins per week) more active participants	51.69 (+/− 18.4)	48.9 (+/− 15.5)
CPM	274.3 (+/−105.1)	265.4 (+/− 91.2)
Trimester 3		
Accelerometer weartime (hrs per day)	12.6 (+/−1.4)	12.6 (+/−1.2)
Accelerometer weartime (valid days)	6.5 (+/−0.9)	6.7 (+/− 0.6)
Light intensity (mins per week)	118.1 (+/− 28.1)	114.4 (+/−26.6)
Moderate intensity (mins per week)	31.7 (+/−17.4)	30.3 (+/− 17.9)
Vigorous intensity (mins per week)	0.8 (+/−0.7)	1.0 (+/−1.0)
MVPA (mins per week)	32.4 (+/− 18.0)	31.3 (+/− 18.6)
MVPA (mins per week) less active participants	27.7 (+/− 9.53)	22.5 (+/− 8.9)
MVPA (mins per week) more active participants	40.53 (+/− 21.19)	40.2 (+/− 21.5)
CPM	238.9 (+/−90.6)	231.5 (+/−98.9)

### PA findings

**Fig. 2:**
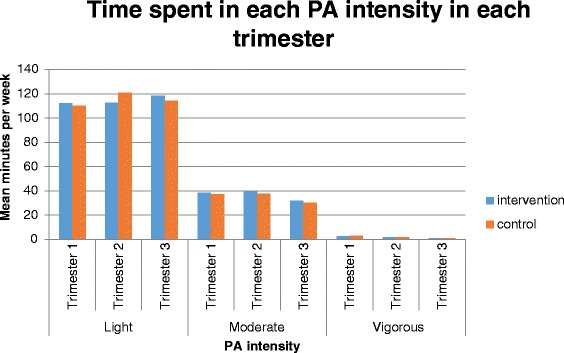
Time spent in each PA intensity in each trimester per group

Accelerometer wear time did not differ between groups in trimester one (*p* = 0.830), trimester two (*p* = 0.506) or trimester three (*p* = 0.915). Mean wear time and time spent in each PA intensity per week are presented in Table 4 and Fig. 2.

**Fig. 3:**
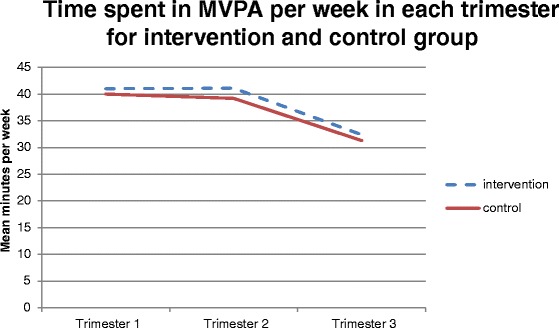
Mean time spent in MVPA per week in intervention and control group in each trimester

Repeated measures ANOVAs indicated that there was a significant main effect of time for moderate intensity PA; F(2, 190) = 16.83, p < 0.001; vigorous intensity PA; F(1.65, 156.42) = 17.34, p < 0.001; MVPA, F(2, 190) = 24.05, p < 0.001 (Fig. 3) and CPM; F(2, 190) = 18.852, p < 0.001, with time in these intensities and CPM significantly decreasing between trimesters one and three (p < 0.001) and trimesters two and three (p < 0.001). There were no significant main effects of time for minutes in light intensity PA; F (1.78, 169.24) = 2.59, *p* = 0.084.

There were no significant time by group interactions for light intensity PA; F(1.78, 169.24) = 2.95, *p* = 0.061; moderate intensity PA; F(2, 190) = 0.011, *p* = 0.989; vigorous intensity PA; F(1.65, 156.42) = 0.585, *p* = 0.526; MVPA; F(2, 190) = 0.07, *p* = 0.936 or CPM; F(2, 190) = 0.225, *p* = 0.799.

**Fig. 4:**
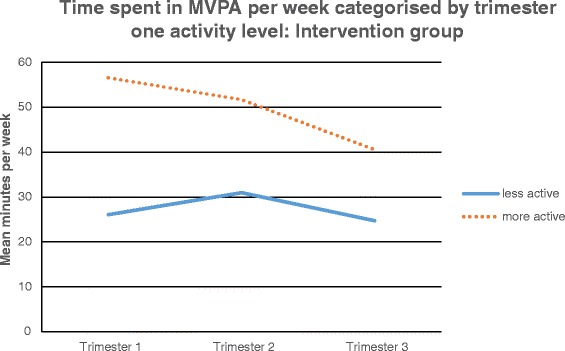
Time spent in MVPA per week categorised by trimester one activity level: intervention group

Within the intervention group the median split of baseline MVPA categorised 24 participants below the median and 23 above. In the control group 25 were below the median and 25 categorised above. In the intervention group, the repeated measures ANOVA indicated a significant main effect of time; F(2,90) = 11.24, p < 0.001 and a significant time by trimester one MVPA interaction; F(2,90) = 6.17, *p* = 0.003 (Fig. 4). This was evident using both the transformed and non-transformed values. This indicates that women who were less active at time one decreased MVPA significantly less over time compared with those who were more active.

**Fig. 5:**
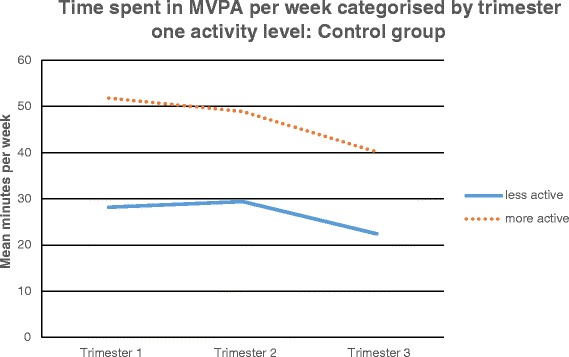
Time spent in MVPA per week categorised by trimester one activity level: control group

In the control group the repeated measures ANOVA indicated that there was a significant main effect of time; F(2,96) = 15.42, p < 0.001 but no significant time by trimester one MVPA interaction; F(2,96) = 1.55, *p* = 0.217, where MVPA in all women decreased over time to the same extent, regardless of trimester one PA (Fig. 5). This was evident using both the transformed and non-transformed values.

## Discussion

### Effect of the intervention

In order to address the need for interventions to facilitate the PA of pregnant women, this study examined whether the established method of PA consultations with application of the HAPA was effective in reducing the magnitude of decline in objectively measured PA in pregnant women. It was hypothesised that those women receiving the intervention of PA consultations would reduce their PA to a lesser extent over the course of pregnancy compared to those in the control group. Results indicated that there were no statistically significant differences between the intervention or control group for PA engagement at any intensity, demonstrating that the intervention delivered did not provide PA benefits greater than usual care alone or attenuate the decline commonly seen in PA throughout pregnancy. However, the sub analysis indicated that those women in the intervention group who were less active in trimester one did not significantly reduce their MVPA throughout pregnancy compared to the significant reduction in MVPA in all other women. This sub analysis finding contributes to support for the use of PA consultations within inactive groups [25–27]. It would therefore appear that the PA consultations delivered in this study were only sufficient in reducing the decline in MVPA in women who were less active in trimester one.

A consideration when interpreting these results is the measurement of PA applied. Of the many PA interventions developed for pregnant women, the predominant method of measurement of PA has been self-report diaries or questionnaires [18] which typically generate biases. Objective measures such as accelerometers reduce this bias but have been applied in very few antenatal PA interventions. Oostdam et al. [43] employed accelerometers to measure activity levels during a structured antenatal exercise intervention. These authors also found no significant difference between the intervention and control groups for MVPA at 24, 32 weeks’ gestation and post intervention and also reported a decline in MVPA from trimester one to three in both groups. As this was also the finding in the APP trial, it could indicate that measurement of antenatal PA via accelerometery offers a more detailed and representative picture of PA behaviours and provides a robust test of intervention efficacy. The application of such a measure in The APP Trial is novel and raises an important methodological issue regarding PA, which has also been raised by Downs et al. [29]. The measurement tool must be considered when assessing the efficacy of PA interventions as it may be influencing the results and subsequent interpretation of intervention success.

In addition to this measurement explanation, when considering the reasons for similarity in PA engagement between the intervention and control groups across the duration of this study, a measurement effect of the accelerometers should be considered. Although previous research indicates that there is little or no effect from of wearing a sealed PA device on free living PA [44, 45], it is possible that participants were more aware of the monitor due to increased awareness of their torso and tightness of the monitor as pregnancy progressed, potentially resulting in a reminder to be active in both the intervention and control. Godin et al. [46] have previously indicated that investigating behaviour through recording or asking questions can influence that behaviour regardless of intervention. In addition, the influence of measuring intentions upon actual behaviour [47], underlines the influence the measurement aspect of this study may have had on behaviour and subsequent similarities in PA between groups.

Another potential explanation of the current findings relates to the intervention intensity. This intervention involved minimal intervention with three face-to-face meetings incorporating behaviour change techniques for the individual to implement on their own, according to their stage of change. This intensity was based on the evidence from previous studies, which have successfully implemented PA consultations with adults [25–27]. Furthermore, the intervention was developed with the potential of being adapted and delivered by health care professionals in standard antenatal care so the clinical feasibility, cost and participant burden had to be minimal. However, the small amount of contact time may not have been sufficient to support women who were more active, to maintain their activity levels as previous antenatal interventions which have been successful in changing behaviour have tended to include more contact sessions. For example, Callaway et al. [48] provided six counselling sessions for women throughout their pregnancy and reported higher levels of PA at 26 and 32 weeks’ gestation. The findings of the APP trial suggest that the reduced amount of contact may have reduced the effectiveness of the intervention. However, through consultation with the health care trust, development of the current study considered the burden upon resources of delivering consultations. In addition, to maximise engagement, the logistical practicalities for women of attending additional consultation sessions (which did not coincide with regular appointments) was considered and influential in the design. Increasing the number of consultations would require additional adjustment to timing and location of delivery of these sessions to reduce participant burden. It is possible that the intervention was not adequate for addressing the physical barriers women often cite as for non-engagement and decline of PA throughout pregnancy. Many researchers have identified size of bump and comfort as being strong determinants of PA disengagement [49–51]. Although this intervention was individually tailored and specific where individual goals were set by participants, it is possible that the physical components of pregnancy were more influential to behaviour, specifically in women who were more active in trimester one. These women may have been engaging in more activities where the physical aspects of pregnancy have greater influence e.g., exercise classes, gym going or cycling and the PA consultation did not adequately support them in finding alternatives to engage in.

The difference in intervention success between less active and more active women indicates potentially different requirements between these two groups. The APP Trial focused on the psychological aspects of behaviour change with no specific mention of the biological, societal or physical influences upon women regarding their PA. It could be hypothesised that the participants classed as more active in trimester one ceased engagement in routine activity due to societal or physical influences for example, approval from others at fitness classes or inability to continue with pre-pregnancy activities due to a growing bump. Partners may not have been as supportive for women to continue with routine activities or women may not have felt comfortable continuing with such activity without explicit permission from their GP [52–54]. These women may require more support and help to identify alternative activities as well as wider education and support from family, friends and peers.

### Patterns of PA

Despite the lacking distinction between groups for PA engagement in trimesters two and three, the pattern of engagement in PA was noteworthy. Means indicate that time spent in light intensity PA increased through the course of pregnancy, when time spent in higher intensities decreased (Fig. 2). This increase can be partially explained by previous literature. Poudevigne and O’Connor [54] recognised that exercise intensity tends to decrease over the course of pregnancy and patterns of intensity also change. The authors reported an overall increase in light intensity exercise and a decrease in moderate and vigorous intensity activities. However, many recent studies assessing or measuring PA during pregnancy only report moderate to vigorous intensity PA since this is the intensity recommended within guidelines. The data regarding an increase in light intensity PA per day provides greater insight into current antenatal PA patterns, measured in a robust manner. However, this finding also raises a further issue of PA measurement using accelerometers for pregnant women. Accelerometers have been used as the gold-standard measure of PA and have been used to validate of self-report questionnaires specifically relating to PA during pregnancy [23], however little is known about a potential change in accuracy of measurement as pregnancy progresses. Specifically, as a woman’s pregnancy progresses she experiences many physiological changes and gradually has more weight to carry. This may increase the relative intensity of certain activities. The accelerometer algorithm cut points applied are absolute and do not take weight into consideration and it may be the case that the cut-points are underestimating intensity. Another consideration could be that women are replacing MVPA with light intensity PA as they are more uncomfortable or worry about causing damage to the baby, a common barrier to PA often cited by pregnant women [14, 16, 17, 49–52].

Finally, this study indicated that moderate, vigorous and MVPA declined the most between trimesters two and three (Fig. 2). This decline has been found in a variety of previous studies investigating patterns of PA throughout pregnancy. Hausenblaus et al. [16] found that pregnant women’s self-reported moderate intensity PA declined during the course of pregnancy. Borodulin et al. [11] reported that overall PA decreased between trimesters two and three; and moderate intensity leisure time PA decreased in each month of pregnancy in a study by Cramp and Bray [15]. The current study demonstrated the same pattern of PA change and indicates this decline in MVPA between trimesters two and three is supported by evidence from a range of different measurements.

### Limitations

This study has a number of limitations which must be taken into consideration when interpreting the findings. Firstly, the sample could be viewed as not representative. The mean CPM per trimester of the participants was similar to data collected in an observational study by Evenson and Wen [55], indicating a similarity in trimester one PA, however, participants of both the APP Trial and the study by Evenson and Wen [55] were predominantly married, white, highly educated and older. Furthermore, there was no measure of pre-pregnancy PA in the current study, therefore it is unclear how representative the sample was in relation to their pre-pregnancy PA levels. This limits the interpretations on the impact of PA consultations in pregnant women.

Secondly, use of acccelerometers to measure PA levels may have limited recording of all activities performed. As the monitor was placed on the trunk of the body, it often fails to accurately pick up upper body movements such as weight lifting, as well as other cardiovascular activities such as cycling. However, development of this technology has resulted in wrist, ankle and arm worn monitors which can help address this limitation of waist worn monitors in future studies [56].

Although the design of the study was a strength, the success and implementation of the intervention and data collection could have been improved if a feasibility or acceptability study had been performed previously. This may have indicated issues with accelerometer wear time or activity and may also have indicated the need for a more intense intervention. A final limitation to consider is the focus of the intervention. The intervention focused on PA which could be categorised into intensities but did not address sedentary behaviours or strength exercises which are both important to the health and wellbeing of pregnant women.

In order to fully understand the potential of PA consultations for addressing the decline in PA throughout pregnancy there are a range of future investigations and developments which could be employed. Recruitment of a larger number of participants with consideration and measurement of their pre-pregnancy PA levels would allow for more robust findings regarding the influence of PA consultations of women with different activity levels. Further investigation is required to find out how these PA consultations could be adapted to benefit women with higher levels of PA at the start of pregnancy as this is clearly an at risk group for reducing MVPA throughout pregnancy.

More research is required to develop an understanding of the impact of using accelerometers to measure PA in pregnant women. Whether they are acting as a behaviour change technique or collecting representative data, it is essential to understand their influence on maternal behaviour. Finally, the sampling bias demonstrated in this and other studies needs to be addressed through more targeted recruitment of a range of women of different ages, education and backgrounds which would provide more generalizable findings.

## Conclusions

In conclusion this study has indicated that PA consultations delivered to pregnant women as part of a theory-based intervention did not alter objectively measured PA compared to standard antenatal care, however, the consultations were beneficial in reducing the decline of MVPA in women with lower PA levels in trimester one. Further work is required to determine if an increase in the intensity of a PA consultation intervention could successfully alter behaviour in pregnant women, specifically those who are more active at the start of pregnancy. In order to improve the accuracy of PA measurement in this group further research and recommendations are required regarding the use of accelerometers with pregnant women, specifically optimum and feasible wear time criteria and intensity cut-points.

## Abbreviations

APP: Active pregnancy profile
BMI: Body mass index
CPM: counts per minute
EM: Estimation maximisation
HAPA: Health action process approach
ITT: intention to treat
MVPA: Moderate and vigorous intensity physical activity
NICE: National Institute for health and care excellence
PA: physical activity
RCT: randomised controlled trial
SD: standard deviation
